# Intralesional doxorubicin as a useful adjunct in the treatment of localized mycosis fungoides tumors with CD30^+^ large cell transformation

**DOI:** 10.1016/j.jdcr.2023.10.013

**Published:** 2023-10-31

**Authors:** Jonathan D. Ho, Jason Thomas, Alicia McNish, Romario Thomas, Janelle Welch, Orchid Dawkins, Rodane Ruddock, Stephanie Smith-Matthews

**Affiliations:** a Section of Dermatology, Department of Medicine, The University of the West Indies, Mona Campus, Jamaica West Indies; b Department of Pathology, The University of the West Indies, Mona Campus, Jamaica West Indies

**Keywords:** CD30, chemotherapy, cutaneous T-cell lymphoma, doxorubicin, intralesional, large cell transformation, mycosis fungoides

## Introduction

Large cell transformation (LCT) in mycosis fungoides (MF), defined by an infiltrate containing ≥25% of large lymphocytes, is associated with poor outcomes and decreased survival.^1^ Its treatment is difficult. Medication access and patient reluctance to commence chemotherapy are challenges in the Caribbean. We describe intralesional conventional doxorubicin (ILD) for treating localized tumors in 2 patients with CD30^+^ transformed MF.

## Case 1

**Fig 1:**
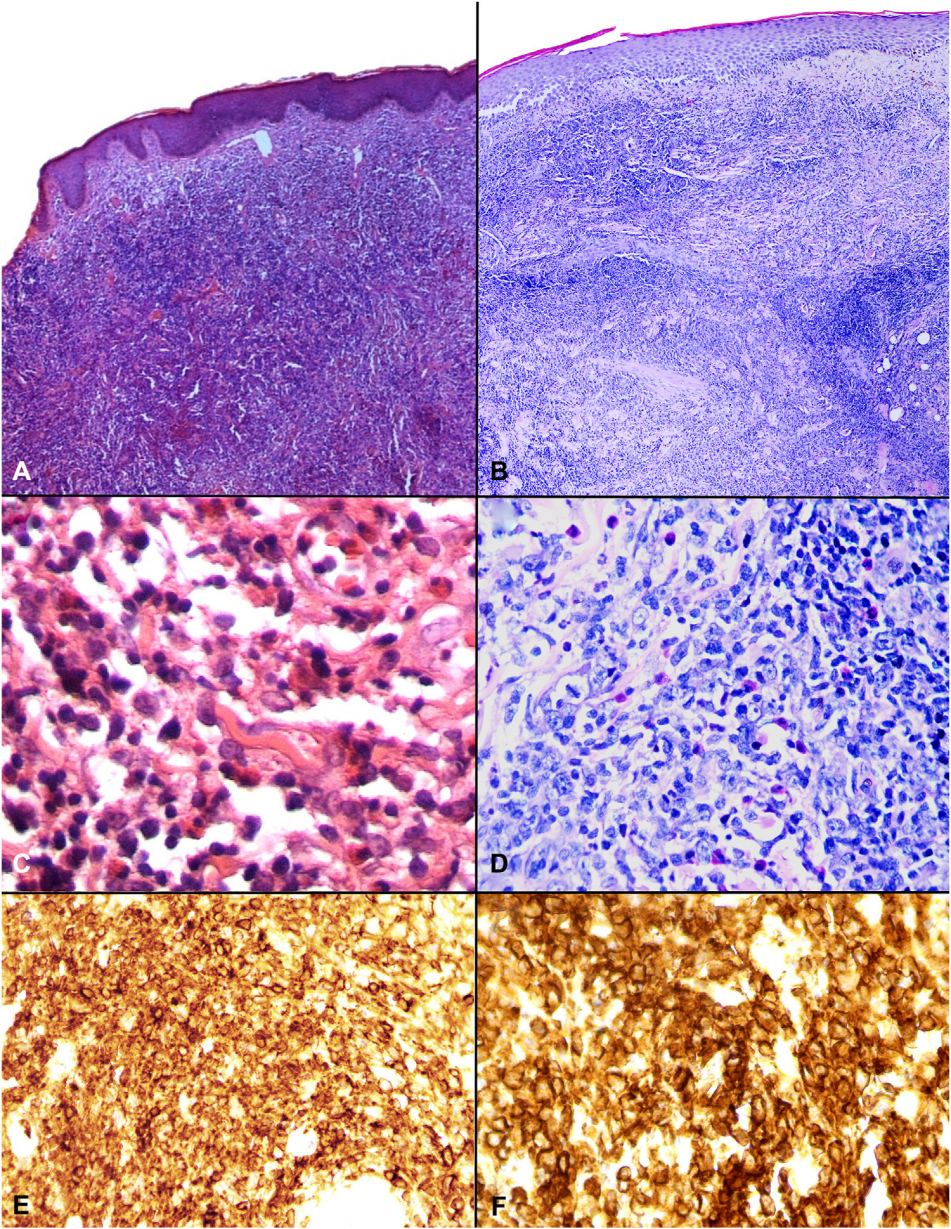
Mycosis fungoides with large cell transformation. Biopsy of tumors from both patients (**A**) 1 and (**B**) 2 reveal a dense pandermal lymphocytic infiltrate. Large cell transformation was noted in both biopsies with cytologic atypia of lymphocytes, large cells comprising >25% of the infiltrate and admixed eosinophils (**C**, patient 1; **D**, patient 2). In patient 1, immunohistochemistry confirmed CD45RO^+^/CD45RA^−^/CD20^−^ T cells with CD30 expression in large forms (not shown), and similarly in patient 2, the malignant lymphocytes were CD45RO^+^/CD3^+^ T cells (**E**, CD45RO shown), (**F**) with CD30 expression. (**A**, **B**, **C**, **D**, **E**, and **F**, Hematoxylin-eosin stain; original magnification: **A,** ×40; **B**, ×40; **C,** ×400; **D**, ×400; **E,** ×200; **F**, ×400).

**Fig 2:**
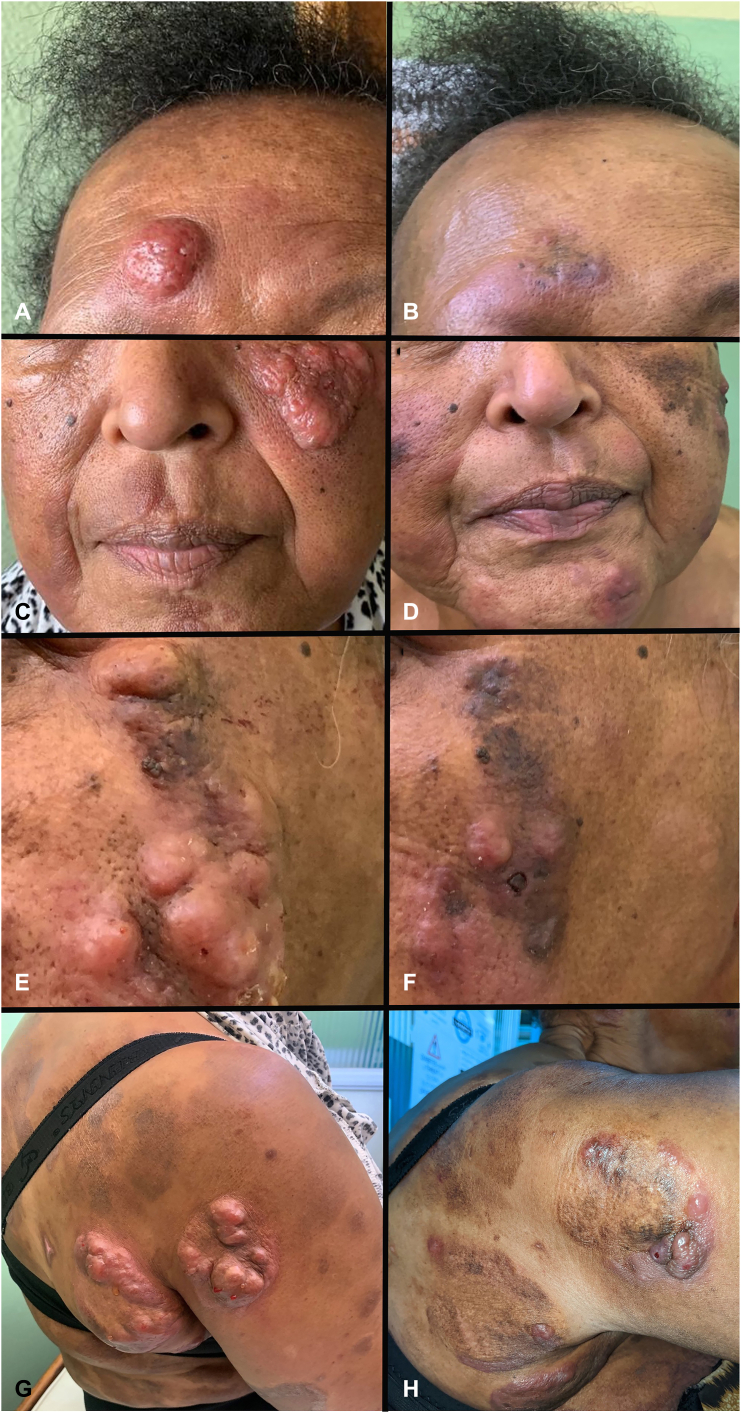
Patient 1. **A**, **C**, **E**, and **G**, Resolution/significant improvement of tumors after intralesional doxorubicin. **B**, **D**, **F**, and **H**, Preinjection images are shown in the left column. Posttreatment in the right column. Although intralesional doxorubicin helps existing lesions, it does not prevent new lesions. **B**, A new tumor arising on the right eyebrow.

A 51-year-old Afro-Caribbean, otherwise well woman with stage IIB (T_3_N_0_M_0_B_0_) MF and CD30^+^ LCT presented for management. She had a 3-year history of untreated patches and plaques. Tumor development prompted referral. Biopsy of plaques revealed Pautrier microabscesses and a lichenoid band of atypical lymphocytes consistent with MF. Tumors demonstrated pandermal lymphocytic infiltration. Large lymphocytes comprised >25% of the cells (Fig 1). Immunohistochemistry revealed CD45RO^+^/CD45RA^−^/CD20^−^ T cells. Large cells expressed CD30. Human T-cell lymphotropic virus (HTLV)-I/II and HIV were negative. Blood counts and blood films were normal, flow cytometry revealed a normal CD4:CD8 ratio, and CT scans of the chest/abdomen/pelvis (CT-CAP) were normal. Clonality studies were locally unavailable. Intravenous (IV) liposomal doxorubicin (20 g/m^2^ = 38 mg) administration on days 1 and 15 and every 28 days thereafter induced excellent response. After 6 cycles, it became locally unavailable and lesions recurred. Because the patient was unwilling to receive further intravenous chemotherapy, oral etoposide therapy (IV formulation taken orally; 100 mg alternate days for 28 days) was commenced. Although plaques responded, tumors progressed (Fig 2, *left column*). Facial tumors caused distress. Considering response to liposomal doxorubicin, we trialed ILD. No concomitant topical therapies were used. After povidone iodine cleaning, doxorubicin powder (50 mg/vial) was reconstituted with normal saline to a 2 mg/mL concentration and injected into tumors on the face and back; 0.1 to 0.3 mL of the solution was infiltrated per injection, 0.5 cm apart, avoiding intravascular injection. Per session, 3 to 4 mL of the solution was injected. Treatment was repeated every other week. After 8 treatments, improvement was noted, with significant flattening of lesions (Fig 2, *right column*). Side effects included pain with injections and hyperpigmentation. No analgesia was used. Although the patient was cosmetically satisfied and discontinued ILD, new lesions occurred within 1 month. The patient agreed to receive IV gemcitabine and showed good clinical response. Unfortunately, the patient died during treatment after contracting a respiratory tract infection.

## Case 2

**Fig 3:**
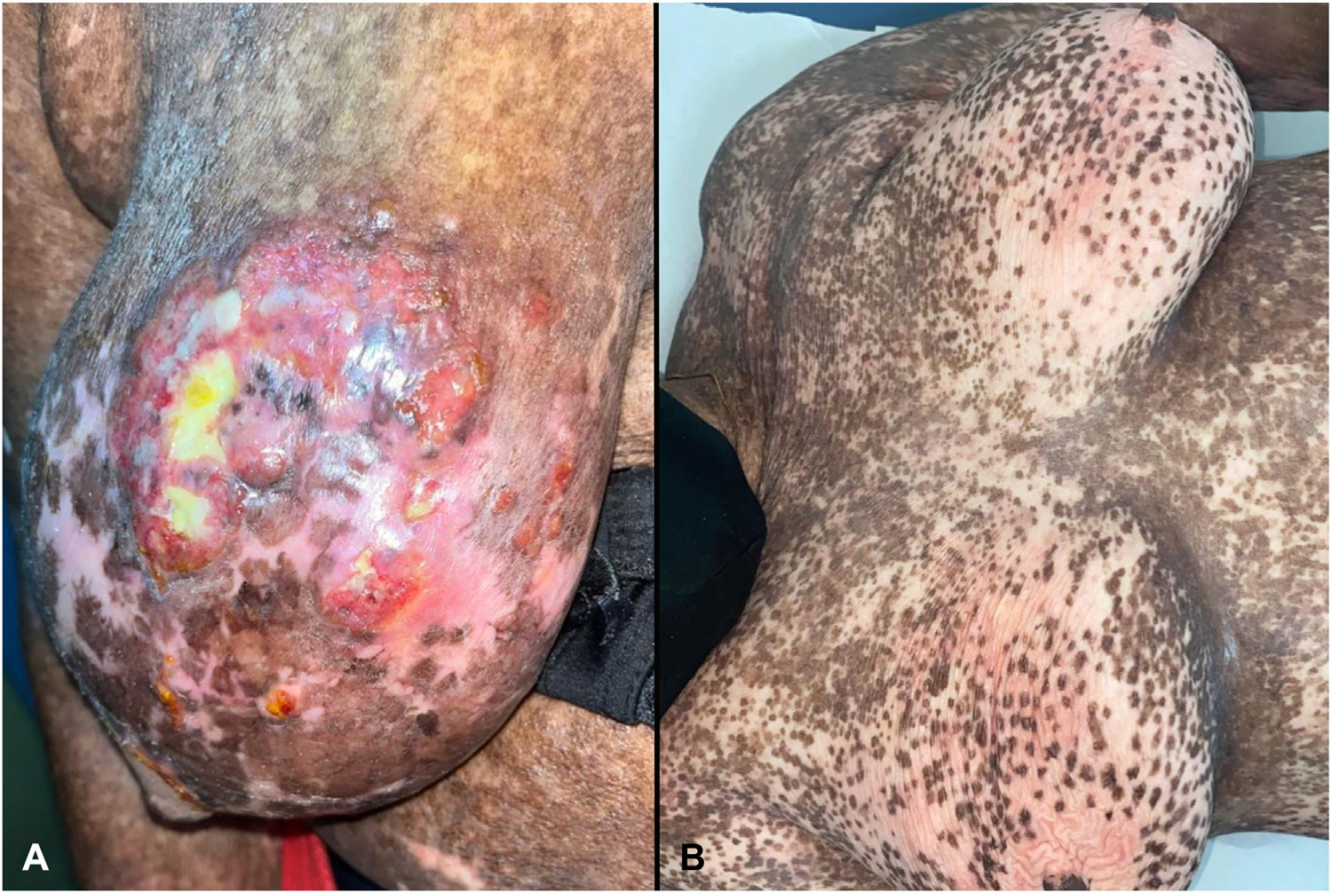
Patient 2. **A**, Significant tumor formation, ulceration, and induration of the breast in mycosis fungoides with large cell transformation. **B**, Near complete resolution of course of intralesional doxorubicin. Patient discontinued phototherapy for her vitiligo, with rapid worsening.

A 67-year-old Afro-Caribbean woman was referred with stage IA (T_1_N_0_M_0_B_x_) MF. Her medical history included rheumatoid arthritis treated with 7.5 mg of methotrexate, vitiligo, and scaly erythematous patches/plaques for many years, diagnosed as chronic spongiotic dermatitis. Persistence despite topical steroids prompted biopsy, which revealed Pautrier microabscesses and a perivascular atypical lymphocytic infiltrate. HTLV-I/II serology was negative. Immunohistochemistry was not performed owing to financial constraints. Nevertheless, the chronicity, patch-plaque progression, and negative HTLV-I/II serology were consistent with MF. We began narrow band UV-B phototherapy and methotrexate 20 mg weekly. One year later, she developed painful ulcerating tumors on the right breast (Fig 3, *A*). Biopsy revealed a pandermal atypical lymphocytic infiltrate, with large forms comprising >25% of the cells (Fig 1). Focal epidermotropism was present. Immunohistochemistry revealed CD3/CD45RO^+^ T cells. CD4 predominated over CD8. Large cells expressed CD30. Although primary cutaneous anaplastic large cell lymphoma was considered and cannot be entirely excluded, pre-existing MF, focal epidermotropism, truncal location, and a typical T-cell phenotype favored CD30^+^ LCT.^2^ Axillary lymph node biopsy revealed dermatopathic changes. CT-CAP was normal (stage IIB T_3_N_1_M_0_B_x_). The patient declined single-agent chemotherapy/radiation and discontinued phototherapy. We offered ILD. No concomitant topical therapy was used. The above protocol was used weekly. Areas of ulceration and induration were infiltrated until healing/decrease in induration was noted. Only indurated/ulcerated areas were treated, and if resolution of a particular area occurred, no further injections were administered. Side effects included injection site pain/pruritus, erythema, and infection of ulcerated tumors (infections with herpes simplex virus and *Serratia marcescens* were treated with oral acyclovir and ciprofloxacin for 1 week). Infection did not preclude treatment of noninfected areas. After 10 sessions, there was significant healing of ulceration and markedly decreased induration (Fig 3, *B*). The patient was satisfied. Although some induration remained, this continued to soften weeks after treatment cessation. Three months later, the lesion had not reulcerated/increased in induration. However, the residual induration and appearance of new tumors on the limbs prompted commencement of gemcitabine chemotherapy.

## Discussion

Management of LCT involves chemotherapy and/or radiation. Liposomal doxorubicin, pralatrexate, gemcitabine, and brentuximab vedotin are recommended by the National Comprehensive Cancer Network guidelines.^3^ In the Caribbean, medication access limits treatment. We also noted patient’s reluctance to commence chemotherapy owing to fear of toxicity. We describe treatment of localized CD30^+^ LCT tumors with ILD. Doxorubicin disrupts topoisomerase-II-related DNA repair and free radical production, damaging malignant lymphocytes.^4^ Local deposits of ILD may exert an effect by mechanistically simulating liposomal doxorubicin whose lipid-encapsulation results in preferential distribution to target tissues rather than cardiac/gastrointestinal deposition.^5^ Although described in Kaposi sarcoma, we could not find reference to ILD use in MF, whereas treatment with intralesional methotrexate and interferon alfa-2 have been reported.^6^, ^7^, ^8^ Regarding cardiac safety, the total dose used over 10 to 16 weeks ranged from 64 to 80 mg. This dose is less than that for a single administration of intravenous conventional doxorubicin (99.56 mg) using a lymphoma protocol at 60 mg/m^2^ (DuBois formula; 60 kg female, average height of 163 cm). With significant risk of cardiomyopathy (∼4%) at total doses of 350 to 550 mg/m^2^, low cumulative dosing and intralesional administration make ILD unlikely to pose significant risk.^9^^,^^10^ Nonetheless, caution is advised in patients with cardiac disease. Leukopenia potentially complicates systemic doxorubicin administration. Both patients presented in this series were on medications requiring routine monitoring (etoposide and methotrexate) and neither developed leukopenia. Localized pain/pruritus/erythema/infection/hyperpigmentation occurred.

ILD may play a role in pauci-lesional disease, tumors in visible areas, and ulcerated/painful lesions. ILD may serve as a bridging therapy for patients awaiting chemotherapy in the absence of systemic disease. Additionally, ILD could decrease medication switching in patients with partial response to a well-tolerated agent. ILD represents an economic and available option. Determining optimal dosing regimens and establishing their safety profile in larger studies are needed.

## Conflicts of interest

None disclosed.
